# Real-Time PCR for Measles Virus Detection on Clinical Specimens with Negative IgM Result in Morocco

**DOI:** 10.1371/journal.pone.0147154

**Published:** 2016-01-26

**Authors:** Touria Benamar, Latifa Tajounte, Amal Alla, Fatima Khebba, Hinda Ahmed, Mick N. Mulders, Abdelkarim Filali-Maltouf, Rajae El Aouad

**Affiliations:** 1 National Institute of Hygiene, Ministry of Health, Rabat, Morocco; 2 Epidemiology Department, Ministry of Health, Rabat, Morocco; 3 Department of Communicable Disease Prevention & Control, WHO/EMRO, Cairo, Egypt; 4 Expanded Programme on Immunization, World Health Organization, Geneva, Switzerland; 5 Microbiology and Molecular Biology Laboratory, Faculty of Sciences, University Mohammed V, Rabat, Morocco; 6 School of Medicine and Pharmacy, University Mohamed V Souissi, Rabat, Morocco; Kliniken der Stadt Köln gGmbH, GERMANY

## Abstract

Since the confirmation of measles cases represents an important indicator regarding the performance of the measles-elimination program, the aim of this study was to evaluate the effectiveness of the routine procedures followed in Morocco for the laboratory confirmation of measles cases. Suspected cases reported between January 2010 and December 2012 were assessed for the timeliness of the sample collection, occurrence of measles clinical symptoms, and the results of the laboratory diagnoses. For 88% of the 2,708 suspected cases, a clinical specimen was collected within 7d of rash onset, of which 50% were IgM-positive and 2.6% were equivocal. The measles symptoms were reported in 91.4% of the cases; the occurrence of symptoms showed a positive association with the serological results (odds ratio [OR] = 2.9883, 95% confidence interval [CI] 2.2238–4.0157). Of the negative samples, 52% (*n* = 116) tested positive by real-time polymerase chain reaction (PCR). These results are in favor of using molecular detection to complement serological diagnosis in the context of measles surveillance approach in Morocco. In addition, the introduction of additional laboratory methods for differential diagnosis is required for the final classification of suspected cases with maculopapular rash and fever in the context of the measles elimination program.

## Introduction

Measles is one of the most contagious human diseases and is caused by the Measles virus (MeV), a member of the family *Paramyxoviridae*, genus *Morbillivirus*. It is considered to be a prime cause of maculopapular rash[1–3]. Globally, 451,756 suspected measles cases were registered in 2014. Measles virus infection causes immunosuppression which leads to severe health complications, including death, especially among young children. In 2013, the WHO estimated the global measles deaths to145,700 [4,5].

Before the introduction of the measles vaccine in 1981, Morocco reported about 120,000 measles cases annually. Measles vaccination coverage increased to 90% in 1996. Despite this high coverage, 1,324 cases were reported that same year [6]. During 23 years a single dose measles vaccination strategy had been adopted. This caused an accumulation of susceptible cases, and continued measles transmission in Morocco. It eventually led to a substantial increase in the number of reported cases, reaching 11,000 in 2003. Therefore, a second routine dose of measles vaccine (MV) was introduced in 2003 for all children at school entry (6 y old) [7,8]. In 2007, however, still 2,248 measles cases were reported. In order to accelerate the reduction in population susceptibility, a mass vaccination campaign (SIA) with the Measles-Rubella (MR) vaccine was conducted in May–June 2008, targeting children aged between 9 months and 14years.

The molecular characterization of MeV isolated from investigated outbreaks has shown circulation of the C2 genotype since 1998 [9] with a co-circulation of genotypes D7 and B3.2 between 2003 and 2005 [10]. Genotype D8 has become the endemic genotype and was circulating in all Moroccan regions between 2005 and 2008. After the 2008 SIA, all isolated strains belonged to the genotype D4, which continued to circulate in Morocco until 2013 [11].

Along with the introduction of this two-dose measles vaccine strategy, a sentinel surveillance network was established in 2007 to complement the outbreak surveillance in order to monitor the impact and effectiveness of immunization activities. Both the Department of Epidemiology and Disease Control and the National Institute of Hygiene coordinate this surveillance network. It involves 15 health centers throughout Morocco, and provides data on the epidemiological and laboratory investigation of sporadic cases.

To avoid the resurgence of measles outbreaks, the World Health Organization (WHO) recommend laboratory confirmation for all sporadic cases and outbreaks in regions reaching or achieving measles elimination; as the incidence is low, the clinical diagnosis becomes inaccurate [12,13]. Therefore, case-based surveillance was introduced in 2010 to further enhance surveillance quality and determine pockets of susceptibility. All sporadic suspect cases and outbreaks are subjected to laboratory confirmation. The diagnosis is based on the detection of MeV-specific immunoglobulin M (IgM) in a single specimen of serum or oral fluid. Molecular investigation for genotyping purposes is conducted by collecting throat swab and/or urine samples.

Since the implementation of sentinel laboratory surveillance in 2007, the investigation of cases over the last decade more than 50% suspect measles cases were discarded. The nation-wide case-based surveillance adopted since January 2010 has shown identical features. In fact, the performance of serological tests depends on the epidemiological setting and the investigation design [12–15].

This study aims to evaluate the added value of the MeV RNA detection by real-time PCR to the diagnosis of measles. For this purpose, we have considered the WHO standard case definition [16], which was adopted in Morocco, the time of specimen collection, and MeV-specific IgM titer. We focused on the cases showing negative IgM results through surveillance data analysis.

## Materials and Methods

Measles is a notifiable disease in Morocco. In January 2010, the Moroccan Ministry of Health (MoH) implemented nationwide case-based measles surveillance as part of its measles-elimination strategy. It required epidemiological investigation and laboratory confirmation of all suspected measles cases. This surveillance involved the 16 Moroccan regions, including 84 hospitals and 2,600 healthcare centers.

### Data and samples collection

Data and samples used in this evaluation are those collected by health professionals during the management of maculopapular rash cases for the purpose of measles surveillance, according to the Moroccan Ministry of Health directives. In the context of this analysis, carried out for public health aims, no additional data or samples were collected from patients. Data and samples were processed anonymously in the National Reference Laboratory for Measles / National Institute of Hygiene which is the MoH reference laboratory.

For this analysis, we have considered the cases detected between January 2010 and December 2012.

### Case Definition

A clinical case of measles, according to the WHO recommendation, is defined as a person with maculopapular rash and fever in addition to cough, coryza, or conjunctivitis, or any person in whom a clinician suspects measles infection [16].

For the purpose of our work, we have considered the case definition in two parts, the symptomatic part, including cases with characteristic measles symptoms, and where a clinician suspected measles.

### Antibody Detection

According to MoH surveillance guidelines for measles, a single set of serum, oral fluid, throat swab, and urine specimens should be collected up on first contact of the suspect case with a healthcare provider.

Sera collected from suspected measles cases were tested for anti-MeV-IgM using the ELISA Anti-Measles-Virus/IgM test kit (Siemens, Marburg, Germany). Oral fluid specimens were tested using the Measles IgM capture EIA (Microimmune Ltd., Hounslow, England). All serum specimens were tested to exclude rubella infection by using the ELISA Anti-Rubella-Virus/IgM test kit (Siemens, Germany). The assays were performed and interpreted according to the manufacturers’ instructions.

### Viral RNA Detection: Specimen Selection

For the viral detection, we considered suspected measles cases from whom, besides serum and oral fluid, throat swabs and urine specimens were collected within 7 d after onset of rash. Specimens were processed upon arrival in the laboratories and stored at–75°C until laboratory diagnosis.

### Epidemiological investigation

A measles case investigation form including enhanced surveillance data (age, gender, place of residence, information about school, case classification, date of disease onset, symptoms, vaccination status, information on similar cases, information on the recent travel, date of sampling, list of collected specimens, indication on the disease outcome) was shipped with each sample.

A real-time PCR test was carried out only if the corresponding serum specimen was negative for IgM.

RNA was extracted using the QIAamp viral RNA mini kit (QIAGEN, Hilden, Germany) according to the manufacturer’s instructions. The RNA was eluted in 60 μl of AVE buffer (RNase-free water containing 0.04% sodium azide) and stored at–75°C until further analysis.

### Real-Time Polymerase Chain Reaction

Real-time PCR assay was used to detect the MeV N gene RNA. Human RNase P mRNA was detected as a control for the integrity of the RNA. Real-time TaqMan-based PCR was carried out using the Platinum OneStepqRT–PCR Kit (Invitrogen, #11732–020) according to the protocol [17].

For the detection of MeV RNA, we used the forward primer (MVN1139-F): 5’-TGG CAT CTG AAC TCG GTA TCA C-3’, the reverse primer (MVN1213-R): 5’-TGT CCT CAG TAG TAT GCA TTG CAA-3’, and the probe (MVNP1163-P): 5’-FAM CCG AGG ATG CAA GGC TTG TTT CAG A BHQ-3’. For the detection of human RNA, we used the forward primer (HURNASE-P-F): 5’-AGA TTT GGA CCT GCG AGC G-3’, the reverse primer (HURNASE-P-R): 5’-GAG CGG CTG TCT CCA CAA GT-3’, and the probe (BHQ1 HURNASE-P): 5’-FAM TTC TGA CCT GAA GGC TCT GCG CG BHQ1-3’. Primers, probes, and control RNA were kindly provided by Dr. Paul Rota (CDC-Atlanta).

The CDC protocol included two internal positive controls containing synthetic MeV RNA (MeV-N3in) and total human RNA: one of low concentration and one of high concentration for the MeV-specific mix, and low- or high-concentration positive control for the non-specific mix. In addition, three negative controls using Nuclease-free water were required: two for the MeV-specific mix and one for the non-specific mix.

Thermal cycling was performed using the ABI 7500 Fast Real-time PCR System (Applied Biosystems, France), under the following conditions: reverse transcription step at 48°C for 30 minutes, activation at 95°C for 5 minutes, followed by 40 cycles of amplification at 95°C for 15 seconds and 60°C for 1 minute. Experiments were set up and analyzed using AB 7500–7500 Fast Real-Time PCR System Software v.2.0.6. The output was the threshold cycle (Ct) value. The interpretation of the results was performed according to the CDC protocol [17].

### Statistical Analysis

Data descriptive analysis was performed using the EpiInfo7 software and data were exported to Microsoft Excel for the creation of the graphs. Cross-tabulations were used to assess the significance of the interactions between considered variables (statistical significance *p* < 0.05).

## Results

During the period January 2010–December 2012, a total of 2,708 suspected measles cases were reported. Measles-specific IgM was detected in 1,348 (50%) cases that had been confirmed as positive, 1,267 (47%) negative, and 70 (2.6%) equivocal. All serum samples had also been tested for rubella in parallel; only 15 (0.6%) cases were positive and 37 (1.4%) equivocal and all these (52) sera were negative for measles. A total of 1345 cases were negative for both measles and rubella IgM.

The overall laboratory confirmation rate was 50%; 45% (492/1,104) in 2010, 58% (578/999) in 2011, and 46% (279/605) in 2012.

### Clinical Symptoms

From a total of 2708 suspected cases, 2,474 (91.4%) showed symptoms defined in the case definition. In the absence of the rash, 1.4% (39) of suspected cases show symptoms encountered during the prodromal period, including fever (100%), cough (79%), coryza (49%), and conjunctivitis (49%). A total of 2,476 (91%) suspected cases were investigated within 10 d after rash onset and 2,021 (82%) cases were investigated within 4 d after rash onset. The date of rash onset was not reported for 5.87% (159/2,708) of the suspected cases.

The concordance rate of the 1^st^ part of th ecase definition ranged between 81% and 96% according to the time sampling.

**Fig 1:**
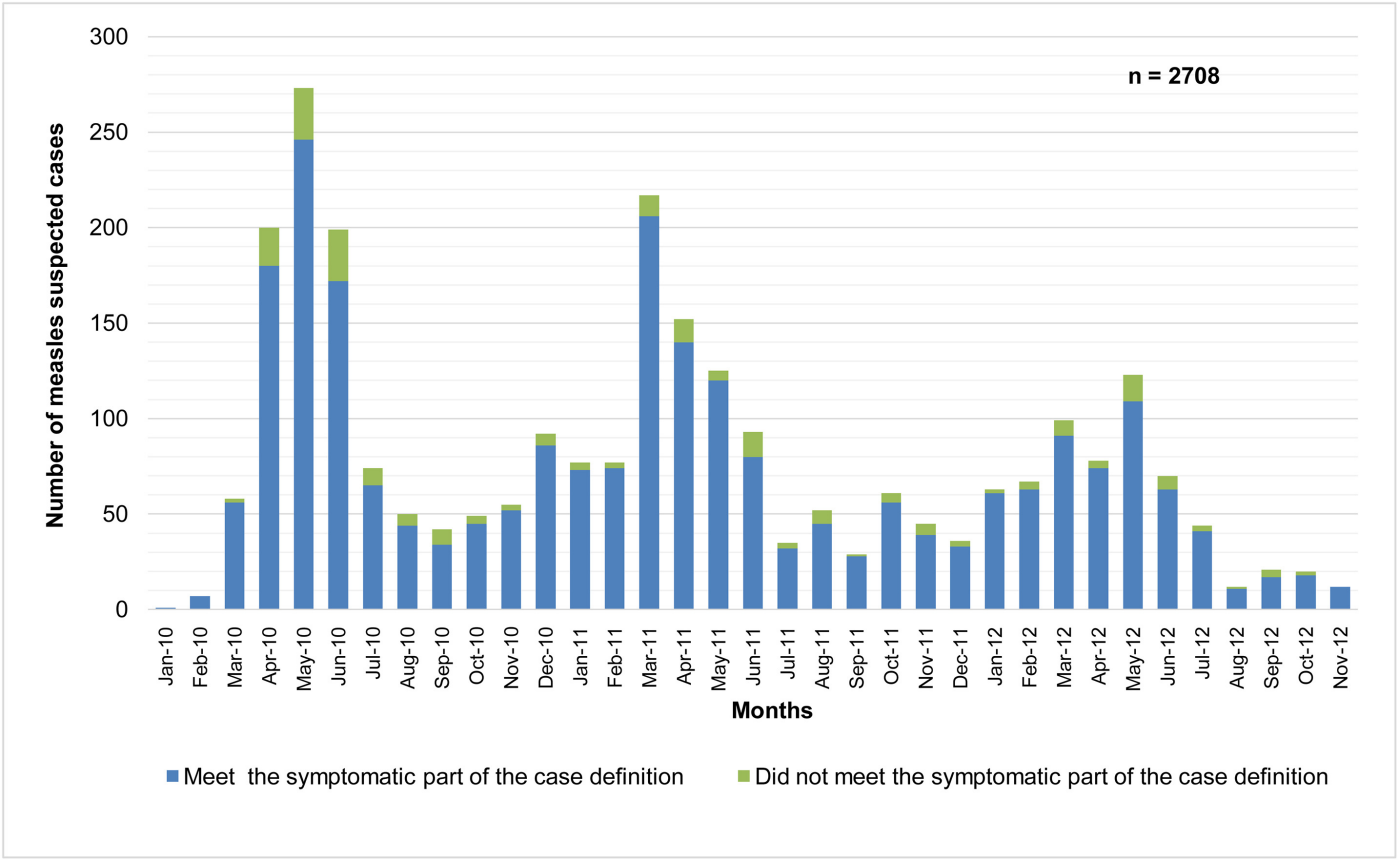
Distribution of suspected measles cases by month and concordance with the 1^st^ part of case definition, Morocco, 2010–2012.

The monthly distribution of suspected cases reveals seasonal epidemics during the spring of each year (Fig 1). Taking into account the concordance rate with the case definition, the monthly distribution does not reveal any significant difference between the months during the epidemic periods and the non-epidemic periods.

### Serological Confirmation

**Fig 2:**
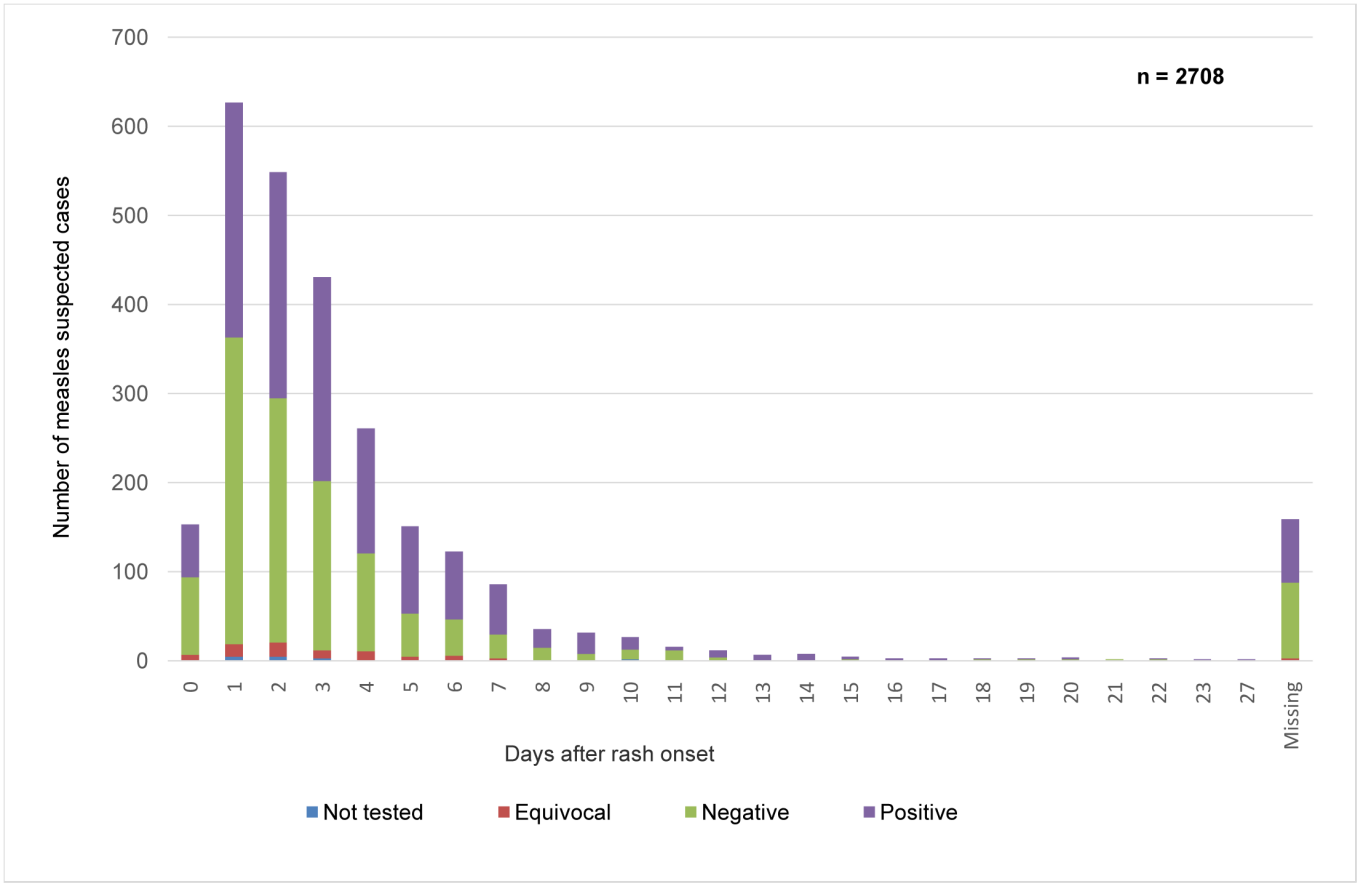
Distribution of suspected measles cases with regard to the time of collection and serological confirmation, Morocco, 2010–2012.

The serological confirmation rate ranged between 39% and 75% for the years 2010–2012 (Fig 2).

The measures of association between response to the symptomatic part of the measles case definition and results of serological confirmation show a positive interaction, with an OR of 2.9883 (95% CI 2.2238–4.0157).

### RNA Virus Detection (Real-Time RT PCR Assay)

Among the negative and equivocal suspected measles cases investigated during the period 2010–2012 within the 10 d after rash onset (1220), a total of 116 (10%); 89 corresponding urine samples and 58 corresponding throat swab samples, were tested using real-time PCR. Ninety nine (85%) responded to the symptomatic part of the measles case definition. Those cases are from 12 regions of the 16 regions in Morocco.

Sixty cases (51.7%) showed a Ct value of less than 40 and were confirmed as positive. The confirmation rate ranged between 20% and 100% with regard to the different regions, while no relationship between the confirmation rate and shipment duration of samples has been noticed. For 31 cases, urine and throat swab samples were tested and showed concordant results for 30 cases (97%).

**Fig 3:**
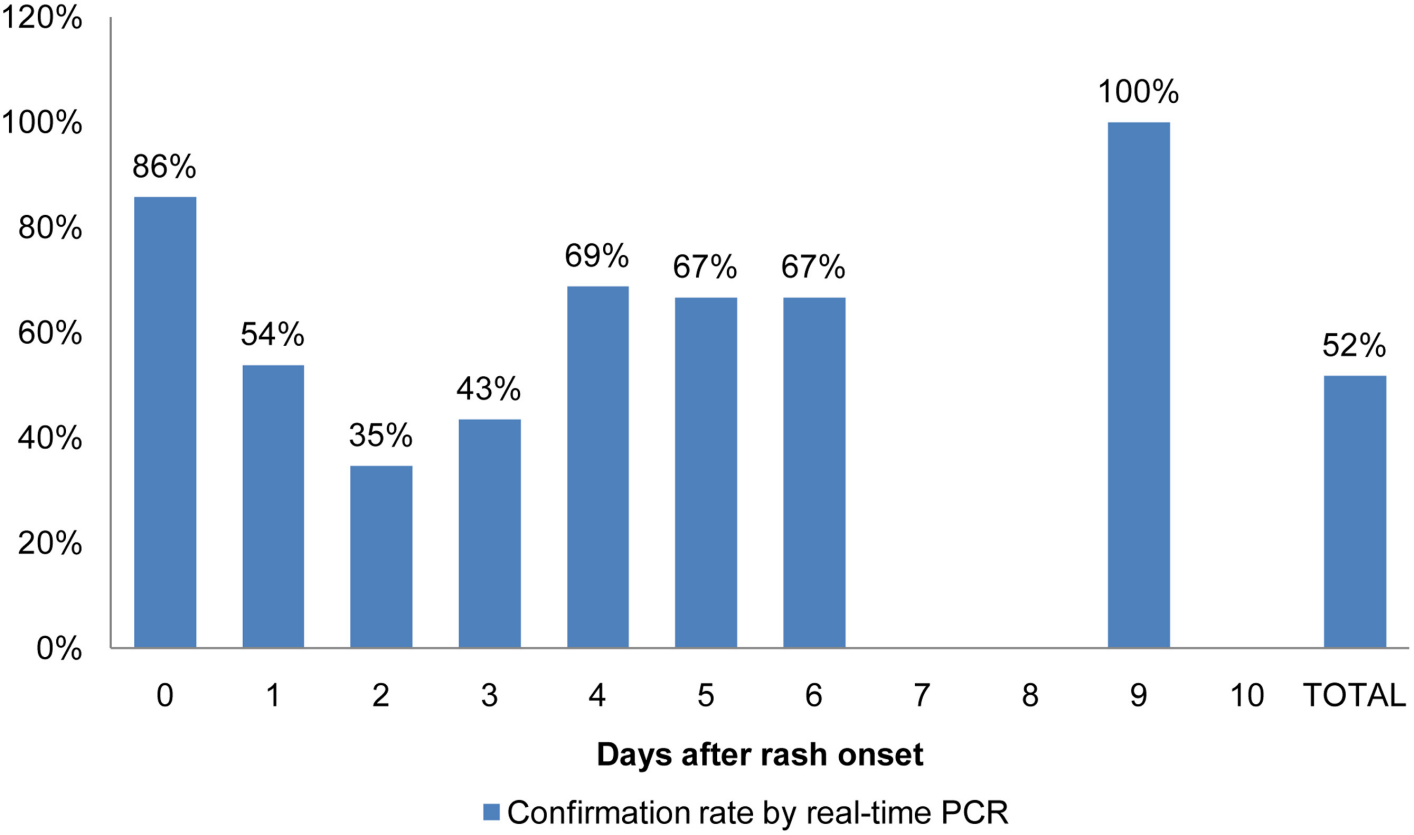
Confirmation rate by real-time PCR test of measles IgM negative cases stratified by the time of sampling, Morocco, 2010–2012.

The MeV RNA detection rate by real-time PCR in urine specimens was 57.3% (51/89) and in throat swabs 50% (29/58). Negative results were found in specimens collected during the first 4 days after rash onset and the highest rate (65%) was observed with samples taken2 d after rash onset (Fig 3).

The measures of association between response to the measles case definition and results of real time PCR confirmation show no significant difference between cases showing symptoms and those enrolled where a clinician suspected measles, with an OR of 0.8049 (95% CI 0.2767–2.3105).

## Discussion

Morocco is still recording a considerable number of suspected measles cases each year, in spite of the high vaccination coverage reported for the routine dose as well as for the mass vaccination campaign in 2008 [18]. Through the establishment of case-based surveillance in 2010, the Moroccan MoH provided a field guide for healthcare professionals including the case definition for measles recommended by the WHO [19]. This study has revealed that almost 9% of cases did not show the characteristic measles symptoms. Indeed, some clinicians, in respect to the second part of the case definition, establish their diagnosis based on a set of symptoms encountered during the prodromal period with a risk to lower the positive predictive value of the clinical diagnosis of measles adopted by the health workers.

In fact, unlike what has been observed in a region with low measles incidence, where it has been shown that physicians are increasingly unable to recognize measles cases [20], clinicians and health workers in Morocco are used to encountering and recognizing measles cases within the first days of rash onset or the prodromal period which may still be negative in ELISA.

The measles case-based surveillance programs implemented around the world as a crucial part of elimination programs have shown a considerable rate of discarded cases [21]. In 2009, from a total of 193,120 samples tested globally for measles IgM, 67% were discarded [22]. Therefore, further laboratory investigation should be conducted, since other infectious agents may cause the same symptoms, such as rubella and parvovirus B19 [17,23].

Methods used for laboratory confirmation are affected by the timing of specimen collection [24–26]. In fact, studies have shown that 30% of serum specimens obtained in the first 72 hours after rash onset reveal negative results, since IgM antibodies are still developing and may be below detectable levels[22,27,28]. Taking into account that case investigation and sampling, in our context, occur early after rash onset, this implies that the number of measles cases reported maybe slightly underestimated based on IgM serology.

In the current study, 65% (CI 63–67%) of samples are collected within four days after rash onset, which may explain the higher rate of Measles IgM negative samples and the concordance between the serology result and the case definition. In fact, by using the real-time PCR, which is known to be highly sensitive and specific [25,29–31], we have demonstrated that 50% of these early reported negative cases are actually positive. Our result did not show a difference in confirmation rate in regard to the time of sample collection within 7 days after rash onset. In addition, the samples tested by the real time PCR were collected from different regions in Morocco, including Rabat region, and showed a comparative rate of positive cases. Knowing that we considered for Real time PCR testing samples with good transportation conditions only, thus, we can exclude any influence of the shipment conditions on the results. The added advantage of collecting urine or throat swab samples, that collection of these samples is not invasive and easy-to-handle [21,32–34]compared to serum samples that are invasive and requires specific conditions regarding transport and conservation to avoid false negatives.

In addition, the result of the Real-Time PCR with regard to the case definition did not show a significant difference between the cases with symptoms and those enrolled based on the clinician suspecting a case. This supports the accuracy of the clinical diagnosis of Moroccan health workers as stated above.

As the elimination program progresses in Morocco, in order to minimize the problem of false laboratory results [17,35,36], it is crucial to strengthen virological investigation by detecting viral RNA.

Nonetheless, our results show that, even for samples collected within 7 days after onset of rash, 50% of the cases were negative for MeV RNA detection using real-time PCR. Since surveillance includes only the rubella infection as a differential diagnosis, which shows a very low incidence, the MoH in Morocco should consider other differential diagnoses, especially those spreading in our region, such as dengue fever[37,38].

In conclusion, with the goal of measles elimination, our results show the accurate application of the case definition by physician in Morocco. In order to overcome the constraints of the field regarding sampling, there is a need for surveillance system reinforcement through two major components: i) the confirmation using virus detection and ii) including additional differential diagnosis.

The previous experience in Morocco with other surveillance programs (HIV, Hepatitis B and C, Influenza) has demonstrated that the financial burden of virological testing could be reduced by two-thirds.
